# Preference for Women's Body Mass and Waist-to-Hip Ratio in Tsimane' Men of the Bolivian Amazon: Biological and Cultural Determinants

**DOI:** 10.1371/journal.pone.0105468

**Published:** 2014-08-22

**Authors:** Piotr Sorokowski, Krzysztof Kościński, Agnieszka Sorokowska, Tomas Huanca

**Affiliations:** 1 Institute of Psychology, University of Wroclaw, Wroclaw, Poland; 2 Institute of Anthropology, Adam Mickiewicz University, Poznań, Poland; 3 Smell and Taste Center, Technische Universität Dresden, Dresden, Germany; 4 Centro Boliviano de Investigación y de Desarrollo Socio Integral, San Borja, Bolivia; Institut Pluridisciplinaire Hubert Curien, France

## Abstract

The issue of cultural universality of waist-to-hip ratio (WHR) attractiveness in women is currently under debate. We tested men's preferences for female WHR in traditional society of Tsimane'(Native Amazonians) of the Bolivian rainforest (*N* = 66). Previous studies showed preferences for high WHR in traditional populations, but they did not control for the women's body mass.We used a method of stimulus creation that enabled us to overcome this problem. We found that WHR lower than the average WHR in the population is preferred independent of cultural conditions. Our participants preferred the silhouettes of low WHR, but high body mass index (BMI), which might suggest that previous results could be an artifact related to employed stimuli. We found also that preferences for female BMI are changeable and depend on environmental conditions and probably acculturation (distance from the city). Interestingly, the Tsimane' men did not associate female WHR with age, health, physical strength or fertility. This suggests that men do not have to be aware of the benefits associated with certain body proportions - an issue that requires further investigation.

## Introduction

For several decades, scientists have been studying the ideals and elements of body attractiveness, such as waist-to-hip ratio (WHR) [1] body mass index (BMI) [2], muscularity [3], breast size [4], leg length (LBR) [5] or height [6]. At the same time, since the beginning of these studies, they have debated whether the obtained preferences are universal or culture/ecology -dependent [7]–[10]. Many of these studies have particularly concerned the attractiveness of BMI (or general body size) and WHR in women in both European and non-European populations [11]–[15]. It remains unclear, however, which values of BMI and WHR are the most attractive and whether the preferences for these body proportions are culturally universal. Below, we present a short review of the research on preference for BMI and WHR, their biological and evolutionary bases, and cross-cultural differences in these preferences.

BMI is predictive of general health in both sexes. The average range of BMI in adults of Western populations is between 18.5 and 25.0, which are the lower and upper limits of the medically normal range for BMI [16]. Individuals who are either underweight or extremely overweight can experience multiple serious health problems such as heart disease, diabetes [17]–[18] and, in the case of women – a higher risk of fertility problems [19]. Despite the benefits associated with average body weight in women, men in North America, Europe and developed Asian countries generally prefer thin compared to average or overweight women [20]–[22]. However, first, there is a population variation in average BMI, with some thin East African pastoralist populations having averages of around 18.5 and – at the other extreme – averages among some Polynesian societies, for example, in Nauru being in an obese range [23]. Second – body weight has large and cross-culturally variable effects on attractiveness [12], [24]–[26], for example in Africa [24], [27] and the Pacific region [28]–[29] where relatively overweight women are preferred.

The question arises as to why preferences for BMI differ to such a large extent. Many studies suggest that the ideal body size may vary across different environmental conditions as a function of resource availability [30]–[33]. For example, Anderson and colleagues [30] reported a negative relationship between regional food availability and attitudes towards fatness in women. More recently, a series of studies showed that men's preferences for women's weight depended on whether they felt hungry [34]–[35]. The direction of these effects was also consistent with the Environmental Security Hypothesis, such that men with fewer resources (less money or food) preferred heavier women [36]. However, the results described above are not very consistent and, moreover, cultural variation in social norms, ideologies, and lifestyle may cause additional variation in men's preferences for women's weight [9].

WHR is another element of attractiveness. In a classic study on physical attractiveness Singh [1], [37] showed that WHR close to 0.7, i.e. a relatively low value, was an important marker of female attractiveness. He also suggested that WHR was a reliable marker of reproductive abilities and women's health [1], [37] and many studies conducted both before and after Singh's observations have confirmed his view: Firstly, that low WHR is a reliable marker indicating that a woman is not pregnant [38]. WHR (resulting from distribution of adipose tissue) is also a result of the activity of male and female sex hormones [39]–[40]. For example, women with lower WHR have higher circulating levels of 17-b-estradiol and progesterone [39], which are predictors of the probability of conception [41]. Furthermore, low WHR might be also reliable marker of probability of conception during in-vitro fertilization [42]. Additionally, the WHR of women decreases (becomes more attractive) during puberty and increases again after menopause [43]. A decrease of WHR has been also linked to the timing of menarche [44]. Finally, low WHR is a reliable marker of a woman's health (e.g., cardiovascular diseases, diabetes) [45].

The question of cultural universality of WHR attractiveness is currently under debate. Probably because of the consistent inverse relationship between WHR and reproduction and health, low values of this trait were suggested to be a relatively universal attractiveness marker. Similar results to the Singh [1] study were obtained for, among others, Europe [46]–[47], Asia [48]–[49], Africa [50]–[51] and New Guinea [14], [52]. Therefore, it can be presumed that men's attraction to women of certain body proportions might indicate a natural and universal preference for healthy and fertile partners.

On the other hand, data obtained in some traditional and remote societies showed that such populations might prefer rather high WHR in women [53]–. Yu and Shepard [56] conducted the first study showing results that were different from the previous culturally universal preferences for low WHR. Among the Matsigenka of Peru living in the Amazon, a WHR of 0.9 was most attractive. Similarly, Marlowe and Wetsman [53], [55] found that the Hadza (hunter-gatherers of Tanzania) perceive a WHR of around 0.9 as the most attractive. However, in a follow-up study using images in profile view in which the buttocks were visible, Marlowe and collaborators [57] found that Hadza men preferred a very low WHR of 0.6.

A few hypotheses have been advanced to explain the discrepancies between the obtained results. Generally, these might be the result of manipulations of the employed stimuli. Low-WHR silhouettes were usually obtained by narrowing the waist (rather than widening the hips) so the observed high ratings of silhouettes with marked waist incision could reflect a preference for low BMI rather than low WHR [2], [22], [47]. At the same time, in non-industrialized populations, women of higher body mass are preferred [30]–[32], [58]. Some scientists, eg. Marlowe [53], explained their results in the following way: "We tested men in a foraging society and found that they preferred high WHRs. We interpret this as a preference for heavier women, which we think should be common where there is no risk of obesity”. A study conducted in the highlands of Papua New Guinea [52] seems to confirm this hypothesis: – Obese women who had undergone micrograft surgery that substantially decreased their WHR were judged by Papuan participants to be most attractive regardless of minor fluctuations in their BMI.

In summary, use of the schematic silhouette drawings similar to those developed by Singh [1] or other researchers was extensively criticized because such drawings are not realistic and it is impossible to determine whether the participants are rating WHR attractiveness, BMI attractiveness or both. Therefore, such stimuli may lead to unreliable results [2], [21]. Additionally, such silhouettes do not seem to be appropriate for use in traditional societies because they are not ecologically valid. In these stimuli the BMI, WHR, skin color, hair color and haircut of silhouettes were different from average women in the tested populations.

In our study we tested the cultural universality of men's preferences for WHR and BMI. We conducted the study among Amazonian society of Tsimane' which practices a traditional economy and is relatively isolated from Western culture and attractiveness standards. To obtain credible results we produced an ecologically valid female silhouette, i.e. body size and shape, skin and hair color typical of Tsimane' women, and digitally modified it in BMI and WHR (independent of each other). In addition, we analyzed the associations between the observed preferences and the men's biological, environmental, and sociocultural characteristics such as age, height, fasting period, TV watching, or distance from the nearest city.

Height and weight are markers of biological quality of an individual, which can be related to malnutrition, particularly during puberty, negative environmental influences, diseases, etc. [59]–[60] as these elements may have an influence the perception of attractiveness [30]–[33]. In our study we also estimated the level of an individual's acculturation, which we understand as the cultural and psychological change resulting from a meeting between different cultures [61]. For this, distance to the nearest city and TV exposure were selected as acculturation measures. Generally, distance from the nearest city is a standard measure of acculturation among the Tsimane' and similar traditional populations [14], [62]. The inclination to watch TV (instead of selecting different ways of spending free time) and time spent watching TV (and observing attractiveness patterns in different cultures) may be considered a good measure of acculturation [63]–[64]. In keeping with other studies on acculturation of the Tsimane' [65], we also investigated the influence of age and wealth on the observed preferences. We also included the variable of a fasting period as previous studies had shown that hunger influenced the perception of attractiveness [34]–[35].

## Methods

### Ethical approval of the study protocol

The study was conducted according to the principles expressed in the Declaration of Helsinki. The study protocol and consent procedure received ethical approval from the Institutional Review Board (IRB) of the University of Wroclaw (Wrocław, Poland) and from the Great Tsimane' Council (the governing body of the Tsimane'). Participants provided informed consent before study inclusion. Owing to the low levels of literacy of the Tsimane', we obtained oral consent for participation and documented this using a portable recorder.

### Participants

The Tsimane' are a native Amazonian society of farmer-foragers. Their population of around 8,000 is distributed throughout approximately 100 villages, most of which are in the area of Beni in northern Bolivia. Because this tribe has been extensively described in the literature [66]–[68], here we only describe their acculturation, which is an important variable in our study.

The Tsimane' were in sporadic contact with Westerners since the seventeenth century, but they avoided permanent contact by moving farther into the hinterlands [69]. Continuous exposure to Westerners dates back only to the early 1950s when the first Protestant missionaries arrived in the area [70]. The highland colonist farmers, cattle ranchers, and logging firms started settling in the region after opening of roads through the Tsimane' territory during the 1970s [67]–[68]. Presently, Tsimane' are the native Amazonian group with one of the largest variation in level of integration to the Bolivian economy, culture and lifestyle [65]–[66], [71]. Some Tsimane' maintain a fairly traditional way of life, ie. fairly isolated, and relying upon shifting cultivation, hunting, fishing, and plant foraging, but others are relatively acculturated, ie. formally educated, sedentary, inhabiting settlements that can be reached by road, and working for wages [72]. These differences might be explained by variation in the proximity of Tsimane' villages to rural towns such as San Borja (population about 35,000). Some villages can be reached in less than two hours' walk from towns, some can be reached by a newly built dirt road, while others can be reached only after several days of travel by canoe.

Our sample comprised a total of 66 men aged between 18 and 50 (*M* = 33.67; *SD* = 11.2). The participants inhabited the region near Maniqui River (dept. of Beni) in the villages of: Campo Bello (n = 22+3 inhabitants of nearby villages who visited their friends or relatives), Maracas (n = 20), Puerto Yucumo (n = 4), Catumare (n = 10), and Anachere (n = 7). We selected these villages because of the highly probable differences in acculturation level. The first village can be reached by car, the next two by a few hours' canoe ride from San Borja, and the last two by at least 2–3 days by canoe. We invited all male habitants aged between 18 and 50 to take part in the study. The level of refusal was low and did not exceed 10–15% per village. In general, Tsimane' villages are very small and so the number of participants indicates that about 80–90% of adult males in the villages of Campo Bello, Maracas and Catumare took part in our study. All participants received a gift (household items worth ∼6 USD) for participating in the series of studies.

### Stimuli

**Figure 1:**
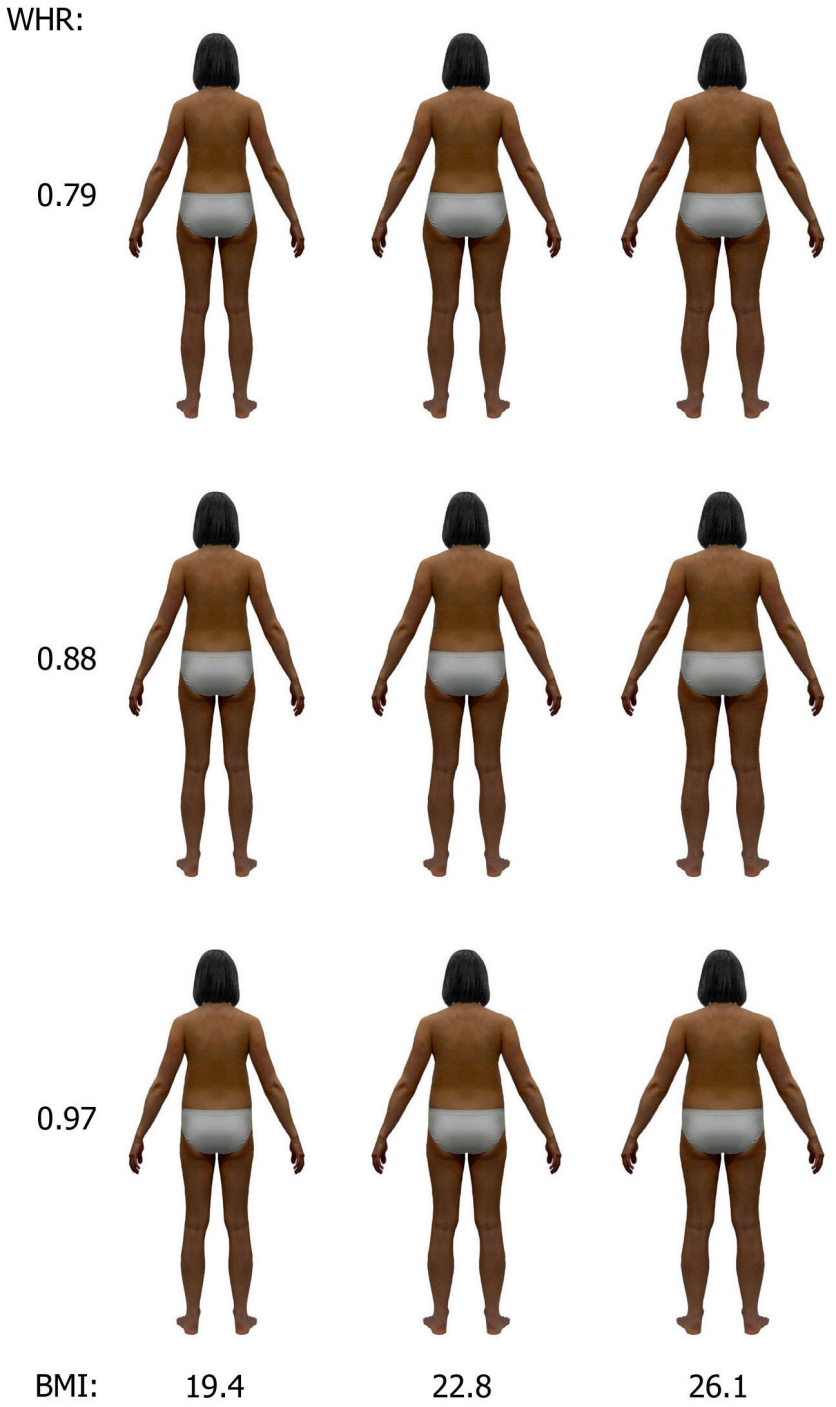
Experimental stimuli consisting of nine female silhouettes of 3 levels of BMI and WHR.

An image of rear-viewed, morphologically average, young European woman was created by K.K. [21] and modified to achieve an appearance congruent with Tsimane' women. In Adobe Photoshop, the brassiere was removed using the Clone tool and the skin and hair color darkened to that typical of the Tsimane'. The resulting silhouette was further independently manipulated for BMI and WHR so as to obtain figures of average, below-average and above-average values for each trait. Reference data were taken from the Tsimane' Panel Data Set 2002–2007 (http://heller.brandeis.edu/sid/tsimane/) [73] for 180 women at age 18–30 years who were not pregnant or breastfeeding during the study (Table 1). The below- and above-average versions of each trait were set to depart from the average by 1.5 standard deviation because this produced silhouettes that differed perceptibly from the average but still fell well within the normal range of the trait. The final set therefore included nine images with three levels of BMI - 19.4, 22.8, 26.1, times three levels of WHR - 0.79, 0.88, 0.97 (Fig. 1).

**Table 1:** Anthropometric data for 180 non-pregnant, non-breastfeeding Tsimane' women at age 18-30.

	*M* (*SD*)	(min–max)
Height (cm)	151.17 (4.71)	(138.1–165.3)
Weight (kg)	52.06 (6.03)	(38.4–77.0)
BMI (kg/m^2^)	22.76 (2.24)	(18.26–31.16)
WHR	0.874 (0.058)	(0.609–1.043)

The manipulations of the silhouettes' BMI and WHR were performed using previously devised and validated methods [21], [74]. Briefly, a change in BMI without influence on WHR was achieved by an appropriate change in overall body width (excluding head). More precisely, a change of *X* percent in body width was assumed to correspond, in real individuals, with the same *X* percent change in body depth, which results in body weight being (1+*X*/100)^2^ of the original weight. A modification of WHR without producing a change in BMI required a simultaneous change in both waist and hip width. Formulas to determine the desired magnitude of changes in waist and hip have been previously developed on the basis of analysis of body shape in Polish women and a three-dimensional digital model of an anatomically correct woman. The magnitude of changes in overall body width (to alter BMI) and in waist and hip width (to alter WHR) was computed with Microsoft Excel and then graphically applied to the initial silhouette using software developed in Microsoft Visual Basic. Images were manipulated by means of warping, a common technique for image distortion used in many studies on attractiveness of faces (e.g., [75]) and silhouettes (e.g., [21]).

### Procedure

A.S. and P.S. conducted the interviews (with the assistance of a translator from the Tsimane' tribe who was fluent in Spanish), with one participant at a time. We showed the participants nine female silhouettes differing in WHR and BMI. The pictures used were 9×13 cm in size and in color. The silhouettes' presentation order was randomized for each participant after which participants answered questions given to each in the order shown below.

Which of the presented silhouettes:

a) is the most attractive (in Tsimane' language: Anic jiyi' jämsi)

b) is the youngest (Mo jäquis)

c) is the healthiest (Mo anic räshsi')

d) is the strongest (Mo anic feryis)

e) seems the best to have children (Mo anic jäm ava'yedyes)

In the next part of the study we measured and interviewed the participants to obtain data on potential predictors of preferences. These included age, height, weight, BMI, body fat, arm circumference (relaxed, tense), wealth, fasting period (time from the last meal), distance to San Borja, TV exposure (hours per week; to calculate a “daily” measure we divided this number by 7). Age of participants was provided by self-report. We measured the height, weight and arm circumference of all participants using an anthropometer, scales (Tanita, model BF680W) and a centimeter, respectively. Body fat was estimated by electronic scales, and BMI value calculated as body weight in kilograms divided by squared height in meters. Fasting period was defined as the number of hours from the last meal.

We assessed the wealth of participants on the basis of answers to a short questionnaire – we asked the participants about the belongings of their families (quantity of fishing nets, chicken, etc.). Afterwards, we multiplied the quantity of given items by their market price. In addition, we controlled for acculturation of the participants by means of two measures: (a) distance from San Borja, where Tsimane' come to buy some commodities, find work, etc. and which is a place of contact of the Tsimane' with Western and Bolivian culture (customs, shops, mass-media, etc.) and (b) exposure to television. Some villages have television (eg. there was a TV set at a teachers' hut in Maracas). Electrical power for the TV was generated from a petrol engine and, because there is no TV coverage, people were only able to repeatedly view the same few DVDs the village had in its possession.

### Analysis

We had 10 variables describing silhouette choices: BMI and WHR of the silhouette chosen as the most attractive, youngest, healthiest, strongest, or of the highest fertility. Each of these variables had 3 levels (low, average, and high BMI or WHR). In cases of random choices each level was expected to have frequency of 1/3. Hypotheses on randomness of choices for a body trait (BMI or WHR) and the judged characteristic (attractiveness, age, health, strength, or fertility), i.e. hypotheses on equality of frequencies of the three categories, was tested with the chi-squared goodness-of-fit test. Equality of frequencies of two specific categories was tested with the binomial test. Associations between choices made in two respects (e.g., attractiveness and health) or made for two body characteristics (e.g., attractive BMI and attractive WHR) were tested with the chi-squared test for independence on 3×3 contingency tables.

To ascertain the dependence of preference for woman's BMI or WHR on men's characteristics, we calculated the Spearman's rank correlation coefficients and conducted multiple regression analyses in the standard and backward stepwise manner. Most of the possible predictors of male preferences were normally distributed. The normal distribution of body fat was achieved by log-transformation, television watching by the transformation of log (*X*+1), and fasting time by the transformation of log (*X*+2). Wealth and circumference of relaxed and tense arm each had one outlying value (4325, 21.5, and 24, respectively); the values were winsorized (substituted by the second most extreme value in the sample) making distributions of these variables normal-like. Age was roughly uniformly distributed in the range of 18–49; however the value of 50 was relatively frequent (*n* = 13), because it was assigned to the elderly men who did not know their exact age and who had estimated that they were about 50. Table 2 provides descriptive statistics for the participants. The database used in this study is available upon request from the corresponding author.

**Table 2:** Descriptive statistics of participants (*n* = 66).

	*M* (*SD*)	(min–max)
Age (years)	33.67 (11.20)	(18.00–50.00)
Height (cm)	166.40 (5.63)	(151.30–180.00)
Weight (kg)	64.27 (6.89)	(46.80–82.70)
BMI (kg/m^2^)	23.21 (2.20)	(16.82–29.37)
Body fat (%)^1^	16.56 (5.56)	(8.00–36.00)
Arm girth, relaxed (cm)^2^	28.06 (1.97)	(24.00–33.00)
Arm girth, tense (cm)^2^	30.94 (1.83)	(26.10–35.00)
Wealth (arbitrary unit)^2^	973.51 (410.56)	(156.25–1837.50)
Fasting period (hours)^1^	4.98 (3.43)	(0.00–16.00)
TV watching (hours/week)^1^	4.02 (7.58)	(0.00–28.00)

1 Data before log-transformation.

2 Data after winsorizing the outlying value.

Statistical analysis was conducted using Statistica StatSoft 8.0 and all reported *p*-values are two-tailed.

## Results

### Attractiveness judgments

Table 3 shows frequencies of BMI and WHR levels in silhouettes chosen as the most attractive.

**Table 3:** Frequencies of BMI and WHR levels in silhouettes chosen as the most attractive, youngest, healthiest, strongest, and of the highest fertility.

	Attractiveness	Youthfulness	Health	Strength	Fertility
BMI level					
1–low	17	42	8	8	17
2–average	11	11	20	21	24
3–high	38	13	38	37	25
WHR level					
1–low	33	25	21	25	24
2–average	18	17	27	19	20
3–high	15	24	18	22	22

A silhouette with high BMI was acknowledged as the most attractive by 57.6% of participants. The chi-square goodness-of-fit test rejected a hypothesis on equality of frequencies of BMI levels (χ^2^ = 18.27, *df* = 2, *p* = .00011). Two-tailed binomial test indicated that the high BMI was chosen more frequently than the average (*p* = .00014) or low BMI (*p* = .0065), while frequencies of the two latter levels did not differ from each other (*p* = .34). In regard to preference for WHR, the most curvaceous body shape (low WHR) was chosen most frequently, by 50.0% men. The chi-squared goodness-of-fit test rejected a hypothesis on equality of frequencies of WHR levels (χ^2^ = 8.45, *df* = 2, *p* = .015). Two-tailed binomial test indicated that the low WHR was chosen more frequently than the average (*p* = .049) or high WHR (*p* = .013), while frequencies of the two latter levels did not differ significantly from each other (*p* = .73).

According to the chi-squared test for independence, the choice of BMI was not related to choice of WHR (χ^2^ = 3.15, *df* = 4, Cramer's *V* = 0.15, *p* = .53).

### Judgments of other characteristics

Table 3 shows frequencies of silhouette choices in respect of perceived youthfulness, health, strength, and fertility.

With regard to BMI levels, the chi-squared goodness-of-fit test rejected a hypothesis on equality of choice frequencies for evaluations of youthfulness (χ^2^ = 27.36, *df* = 2, *p* = .000001), health (χ^2^ = 20.73, *df* = 2, *p* = .00003), and strength (χ^2^ = 19.18, *df* = 2, *p* = .00007), but not fertility (χ^2^ = 1.73, *df* = 2, *p* = .42). Silhouettes with high BMI were perceived as the most healthy and strong, while those with low BMI were regarded as the youngest.

On the other hand, choices of silhouettes in respect of WHR were randomly distributed irrespective of the choice criterion: youthfulness (χ^2^ = 1.73, *df* = 2, *p* = .42), health (χ^2^ = 1.91, *df* = 2, *p* = 0.38), strength (χ^2^ = 0.82, *df* = 2, *p* = .66), or fertility (χ^2^ = 0.36, *df* = 2, *p* = .83).

### BMI attractiveness and other evaluations

According to the chi-squared test for independence, BMI of the silhouette chosen as the most attractive was not related to BMI of the silhouette selected as the youngest (χ^2^ = 2.93, *df* = 4, Cramer's *V* = 0.15, *p* = .57), healthiest (χ^2^ = 2.99, *df* = 4, Cramer's *V* = 0.15, *p* = .56), strongest (χ^2^ = 8.99, *df* = 4, Cramer's *V* = 0.26, *p* = .061), or of the highest fertility (χ^2^ = 2.37, *df* = 4, Cramer's *V* = 0.13, *p* = .67). However, the relationship for the perceived strength was close to the conventional threshold of statistical significance, and the Spearman's rank correlation coefficient indicated that the direction of the association was positive (*R* = .20). This tentatively suggests that a man's preference for women's BMI is related to how he perceives the physical strength in female silhouettes.

### WHR attractiveness and other evaluations

According to the chi-squared test for independence, WHR of the silhouette chosen as the most attractive was not related to WHR of the silhouette selected as the youngest (χ^2^ = 0.74, *df* = 4, Cramer's *V* = 0.08, *p* = .95), healthiest (χ^2^ = 4.34, *df* = 4, Cramer's *V* = 0.18, *p* = .36), strongest (χ^2^ = 0.83, *df* = 4, Cramer's *V* = 0.08, *p* = .93), or of the highest fertility (χ^2^ = 2.99, *df* = 4, Cramer's *V* = 0.15, *p* = .56).

### Determinants of preference for BMI

As assessed with the Spearman's rank correlation coefficient, the preference for BMI was associated with the judge's age (*R* = .46, *t* = 4.16, *p* = .0001), height (*R* = −.40, *t* = 3.51, *p* = .0008), body fat (*R* = .27, *t* = 2.28, *p* = .026), and distance to the city (*R* = .37, *t* = 3.22, *p* = .002); p-values for remaining variables were above 0.2.

To determine a unique and independent effect of each predictor on preference for BMI, we conducted a standard multiple regression of the preference for BMI on the set of potential predictors. The model was significant (*R* ^2^ = 40%, *F*_11,54_ = 3.27, *p* = .002) and indicated a significant effect of age (standardized beta (β) = 0.37, *p* = .010) and distance to the city β = 0.33, *p* = .011). The same predictors were established when the regression analysis was carried out in a backward stepwise manner (age: β = 0.43, *p* = .00008, distance to the city: β = 0.37, *p* = .0007).

**Figure 2:**
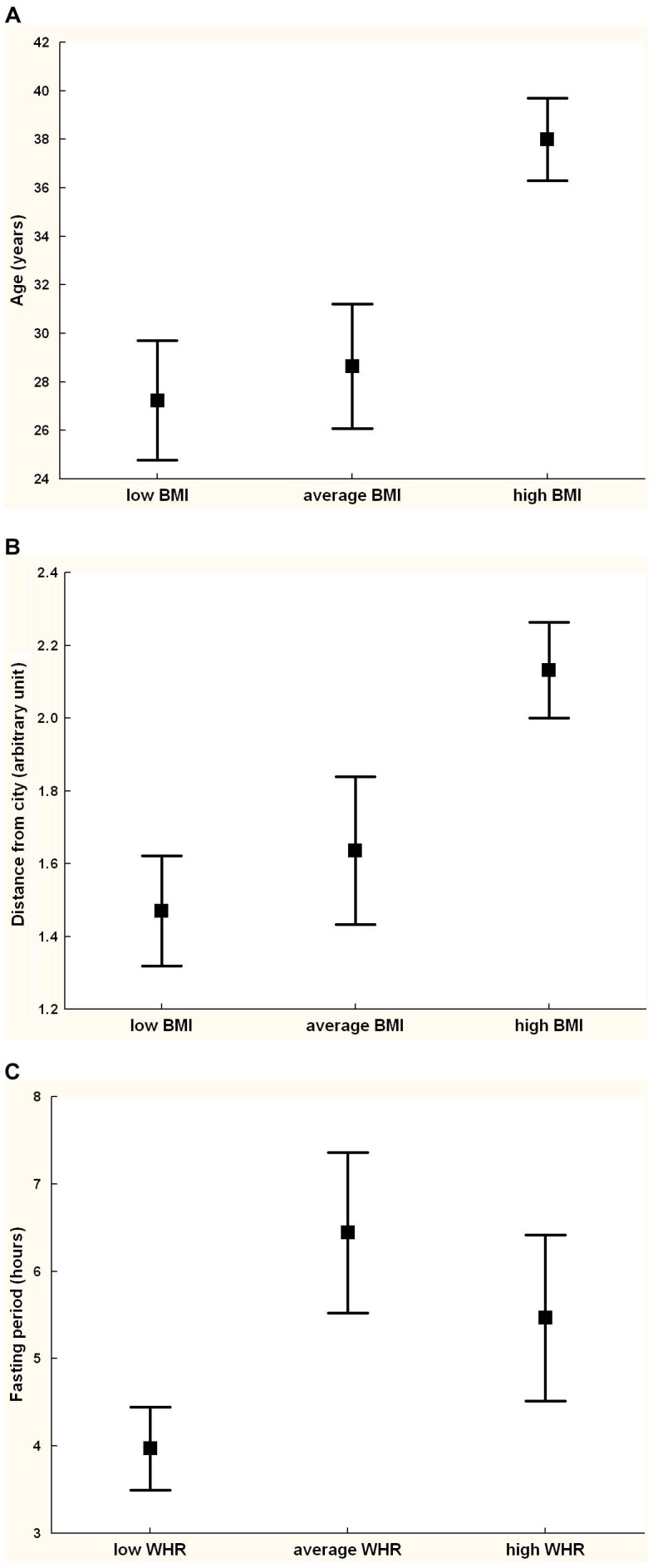
Age (A), distance from city (B), and fasting period (C) of men who chose low, average, and high BMI or WHR as the most attractive.

To ensure that these results were not biased by the low number of possible values for the dependent variable (3 BMI levels), we conducted a multiple logistic regression with the same set of predictors and the dependent variable taking two values, 1 if the high BMI was chosen, and 0 if otherwise. The judge's age and distance to the city again proved the only significant factors, irrespective of whether the analysis was performed in the standard (age: odds ratio (*OR*) = 1.12, *p* = .009, distance to the city: *OR* = 3.78, *p* = .015) or backward stepwise manner (age: *OR* = 1.12, *p* = .0004, distance to the city: *OR* = 3.94, *p* = .002). See also Fig. 2.

All results, and particularly the effect of age, remained very similar when 13 men with exact age not known were omitted in the analyses (not reported here).

### Determinants of preference for WHR

The judge's fasting period was the only characteristic that significantly correlated with his preference for WHR (Spearman's *R* = .25, *p* = .042); p-values for remaining variables were above 0.08. Note, however, that the significant effect for fasting period would not survive any correction for multiple testing.

A standard multiple regression of preference for WHR on the set of potential predictors was non-significant (*R* ^2^ = 14%, *F*_11,54_ = 0.81, *p* = .63) as were all predictors (all *p*s>.09). When this analysis was carried out in a backward stepwise manner, all predictors were removed from the model. We also conducted a multiple logistic regression with the same set of predictors and the dependent variable taking two values, 0 if the low WHR was chosen, and 1 if otherwise. Now, the judge's fasting period proved the only significant factor, irrespective of whether the analysis was performed in the standard (*OR* = 5.46, *p* = .025) or backward stepwise manner which left in the model only one predictor, the fasting period (*OR* = 4.45, *p* = .021). See also Fig. 2.

## Discussion

In our study, conducted among the Amazonian society of the Tsimane', we tested whether the preferences for WHR and BMI are universal. Generally, we found that Tsimane' men prefer high BMI and low WHR, attribute health and strength to the silhouettes of higher BMI and younger age to the silhouettes of lower BMI, and do not associate any of these characteristics with WHR. We showed that the preferences for BMI depended on age and distance from San Borja, and that preferences for WHR depended on the satiety level.

The preference of Tsimane' men for BMI higher than the average BMI in their population is consistent with previous observations that relatively high body mass is more valued in populations that may experience problems with food availability [30]–[33]. In addition, our results regarding attractiveness of WHR seem to be consistent with the results of some previous studies [48], [51], [57]. The Tsimane men preferred silhouettes of WHR lower than the average WHR for Tsimane women. According to our knowledge, this study is the second existing work showing preferences for high BMI and low WHR. Sugiyama [54] obtained similar results among the Shiwiar of Ecuador (who also inhabit the Amazon region). Our results show also that the preferences for high BMI do not have to be associated with preferences for high WHR – these preferences did not correlate in our sample.

We found, however, other variables correlating with preferences for BMI. Higher BMI was preferred by older men and by men living in villages farther from the town of San Borja. In the initial analyses we also observed that the preferences for BMI correlated with participants' own height and fat level, but these results turned out to be non-significant in further multivariate analyses. We can hypothesize that age and distance from the town are elements of acculturation. On the other hand, watching TV did not correlate with preferences for BMI (*R* = −.04, *p* = .74). Absence of this correlation shows that the sole factor of watching television is not enough to change the preferences for body shape (despite previous hypotheses; [76]).

We also hypothesize that distance from San Borja and contact with the market economy might influence the living conditions of the Tsimane' [77]. In our study body height, which is known to reflect the life conditions the person experienced in childhood [78], negatively correlated with the distance from San Borja (*R* = −.29, *p* = .019) and age (*R* = −.31, *p* = .012). The differences in height between the old and the young people and people living closer to San Borja may be the result of the different environmental conditions in which they grew up. In our study body height strongly correlated with preferences for BMI (*R* = −.40, *p* = .0008) but ceased to be a significant predictor when age and distance from San Borja were introduced into the regression analysis. This suggests that the influence of male body height on this preference is mediated by a man's age and place of residence. We might therefore presume that ecological conditions specific for a village at some time modify both a man's stature (at childhood) and his preference for BMI. For example, distance from the town might correlate with food availability and/or level of physical workload of women, and the effect of age might be associated with the fact that in the past men had more problems obtaining food for their families, their wives had to work more than now and women of higher BMI were perceived as better nourished and stronger. Our hypothesis is supported by the fact that child and adult survivorship and life expectancy increased substantially with time and proximity to San Borja due to, presumably, temporal and regional variation in nutritional status and medical care [79]; in addition, growth stunting tends to be more frequent in the more distant villages [79]. We cannot exclude that the associations of height with age and distance to the city have genetic foundations (due to, for example, a selective migration) and the impact of age and the distance on preference for BMI results from acculturation, though the abovementioned hypothesis of variation in living conditions seems the more parsimonious and less speculative.

On the other hand, a few previous systematic studies conducted among Tsimane' showed that many nutritional indicators do not seem to vary in relation to the integration with market and acculturation [80]. We do not have reliable data about the Tsimane' in the past, but the members of TAPS research group have not observed dramatic changes in living conditions of the Tsimane' in the last 20 years, despite there having been some changes (e.g., more government schools). Finally, two cross-sectional waves of data collected from 861 Tsimane' showed no clear trends in their height [81], which might suggest that our hypotheses could be erroneous. In summary, our results support the hypothesis of life condition influencing the preference for BMI whereas other studies on Tsimane' challenge it. We also cannot dismiss the hypothesis of acculturation even though it received no clear support from our data.

At the same time, the preferences for WHR seem to be less dependent on the previously mentioned biological, ecological (except for hunger level) and cultural factors than BMI preferences. The results of our study suggest that whereas BMI attractiveness seems to be changeable, attractiveness of certain WHRs is much more stable and cannot be easily modified. This might result from strong relationship between the WHR and health and fertility [39]–[40], [43], [45]. Interestingly, we found that participants' satiety was related to their preferences for WHR, but not to the preferences for BMI. In three previous studies conducted among Western populations [34]–[35], [82] it was shown that hunger slightly influences the preferences for weight or BMI/body weight, but this effect was observed for WHR in only one of two studies that tested it [82]. In our study only the preference for WHR was related to hunger level. Obviously, the Tsimane' seem to eat less frequently than an average participant from Western populations (some Tsimane' participating in the study reported eating their last meal a day before) and this could make our results regarding WHR the more salient. At the same time, lack of correlation between time from last meal and BMI preferences suggests that hunger might induce the preferences for women having more adipose tissue in the stomach area (not more fat in general) because fat in this area can easily be transformed to energy in metabolic processes when food is not available. Thus, current hunger in men might make them prefer women who have energy resources which might be easily utilized during periodic hunger, and not women who have more adipose tissue created for different aims, e.g., gluteofemoral fat for a possible future child [44].

Another possible explanation for the observed results is that low WHR is a typically feminine characteristic – women generally have lower WHR than men [83]. Men probably notice this and perceive lower WHR as more feminine. However, if that was the case with Tsimane' men we would expect that they would associate lower WHR with other feminine characteristics such as lower strength and higher childbearing capability, yet we did not observe such relationship.

Although men perceived low WHR as attractive, they did not associate it with any of youth, health, strength, or fertility. This result was unexpected because WHR is known to be positively related to age [83] and negatively to health [45] and fertility [39]–[40], and WHR correlated positively with age in Tsimane' non-pregnant woman aged 18–50 (*r* = 0.20, *N* = 1007, *p*<0.001, according to TAPS data). The question arises as to why the men actually preferred the low WHR. We hypothesize that men (including Tsimane') do not have to be aware of the relationship between WHR and health or fertility – their preferences might have evolved as a consequence of adaptive benefits related to selecting women of this silhouette as partners [84].

Yet another possibility is that Tsimane' associate low WHR with absence of pregnancy. However, although a female's WHR increases during pregnancy due to the belly's progressive protrusion, transverse WHR undergoes but a small change [38]. It is therefore improbable that men can infer the presence of pregnancy from such an uncertain cue as the transverse WHR if they can do so much more reliably on the basis of belly shape. Also, Schützwohl [85] argue that people prefer low WHR not because they associate it with a low probability of pregnancy, but that male preferences for low WHR and the hypothetical decrease of sexual attractiveness of pregnant women seem to be based on different psychological mechanisms.

A problem associated with the interpretation of some of our results is that one of our questions was "which of the presented silhouettes seems the best to have children". We used this question because other, more precise formulations were not understandable by the participants. However, this question could be understood as both a question about fertility (i.e. current fecundity) and reproductive potential (pertaining to a lifetime capability). These two variables are not exactly the same [86]–[87].

In the present study the preferences for BMI tended to correlate with perceived strength. This suggests that attractiveness in traditional populations could be associated with a capacity for hard work, which has been suggested previously [88]. However, our study does not present a very strong argument for this hypothesis (because the correlation was only marginally significant), which also suggests the possibility that the sources of preferences for BMI are not conscious.

In summary, the results of our study support the hypothesis suggesting that WHR lower than the average in a given population is preferred universally, and is independent of ecological and cultural conditions. Some of previous studies have also shown preferences for high WHR in traditional populations, but these were not properly controlled for body mass: The stimuli we employed enabled us to overcome this problem. Furthermore, since our participants preferred silhouettes of low WHR, but high BMI, this might suggest that these previous results could be an artifact related to the employed stimuli. However, to be certain that previous stimuli may have biased the outcomes we would have needed to conduct our study in the same populations as the previous with the old and new stimuli sets.

We have also shown that preferences for female BMI might be changeable and dependent on many factors. Interestingly, the Tsimane' men – who found low WHR attractive – did not associate it with perceived age, health, physical strength or the reproductive potential of women. This suggests that the sources of preferences for certain body proportions might not be conscious, but this issue requires further research which includes qualitative measures such as interviews or observations.
